# Comparative Impact of Vitamin D Deficiency on Immune Function and Disease Susceptibility in Pediatric Versus Adult Populations

**DOI:** 10.7759/cureus.101582

**Published:** 2026-01-15

**Authors:** Amara Tahira, Muhammad Usama Tahir, Sana Sharif, Husnul Hayat, Misbah Arshad, Omair Mazhar, Samreen Amjad, Aneela Zareen

**Affiliations:** 1 Family Medicine, Saleena Children Clinic, Lahore, PAK; 2 Family Medicine, Islam Medical Center, Lahore, PAK; 3 Pediatrics, Avicenna Medical College and Hospital, Lahore, PAK; 4 Pediatrics, Bhatti International Hospital, Kasur, PAK

**Keywords:** adult population, disease susceptibility, immune function, pediatric population, vitamin d deficiency

## Abstract

Background: Vitamin D deficiency is a common global health problem and has been widely studied in relation to immune function and infectious diseases. Vitamin D receptors are expressed on multiple immune cells, suggesting a potential association between vitamin D status and immune-related parameters. However, comparative observational data examining age-related differences in immune markers and infection patterns among vitamin D-deficient individuals remain limited.

Objective: The study aimed to describe and compare associations between vitamin D deficiency, selected immune markers, and reported infection patterns in pediatric and adult patients.

Methods: This comparative cross-sectional observational study was conducted at a tertiary care hospital. Pediatric (1-18 years) and adult (≥19 years) patients with vitamin D deficiency were included. Serum 25-hydroxyvitamin D levels, immune markers (total lymphocyte count, immunoglobulin levels, C-reactive protein, and erythrocyte sedimentation rate), and reported respiratory and gastrointestinal infections over the preceding six months were assessed. Unadjusted comparisons between pediatric and adult groups were performed using appropriate statistical tests, and associations between vitamin D levels and immune markers were evaluated using correlation analysis.

Results: A total of 120 participants were included, comprising 60 pediatric and 60 adult patients. Pediatric participants had lower measured serum vitamin D levels, lower total lymphocyte counts, and lower IgG levels compared to adult participants. Higher reported frequencies of respiratory and gastrointestinal infections were observed among children. A moderate positive correlation was observed between serum vitamin D levels and total lymphocyte counts across both age groups.

Conclusion: Vitamin D deficiency was associated with differences in selected immune markers and reported infection patterns between pediatric and adult patients. These findings represent descriptive associations observed at a single time point and do not establish causality or directionality. Further longitudinal and interventional studies are required to clarify temporal relationships and clinical significance.

## Introduction

Vitamin D is a fat-soluble steroid hormone that is critical to calcium and phosphate homeostasis, skeletal development, and overall metabolic well-being. Over the last few years, there has been growing interest in its extra-skeletal roles, particularly its association with immune function and susceptibility to disease. Vitamin D receptors are present on a wide range of immune cells, including T lymphocytes, B lymphocytes, macrophages, and dendritic cells, suggesting a role for vitamin D in immunomodulatory processes [1].

Vitamin D deficiency is a global health issue affecting both children and adults, with higher prevalence reported in underdeveloped and developing regions. Deficiency may occur due to limited sun exposure, dietary inadequacy, malabsorption, darker skin pigmentation, and lifestyle-related factors. Periods of rapid growth in childhood are associated with increased nutritional requirements, while in adults, age-related physiological changes and comorbid conditions may influence vitamin D status [2].

Vitamin D status has been widely studied in relation to immune system functioning. Adequate levels have been associated with enhanced innate immune responses through stimulation of antimicrobial peptides such as cathelicidin and defensins, as well as modulation of adaptive immune responses that may limit excessive inflammation. Several observational studies have reported associations between vitamin D deficiency and respiratory tract infections, autoimmune diseases, and chronic inflammatory conditions; however, these findings remain largely associative and context dependent [3].

Children represent a distinct population in whom the immune system matures progressively with age. Vitamin D deficiency in pediatric populations has been associated with increased frequency of infections, asthma exacerbations, allergic diseases, and altered immune responses. Given the importance of immune development during early life, variations in immune parameters observed in childhood may have implications for long-term health, although causal relationships cannot be established from cross-sectional observations [4].

In adults, vitamin D deficiency has similarly been observed alongside altered immune and inflammatory markers, particularly among individuals with chronic conditions such as diabetes, cardiovascular disease, and autoimmune disorders. These findings suggest that vitamin D status may coexist with immune dysregulation in adult populations, though the direction and magnitude of this relationship remain uncertain in observational settings [5].

Although the relationship between vitamin D status and immune-related outcomes has been explored extensively, comparative observational data examining how vitamin D deficiency is associated with immune markers and infection patterns across different age groups remain limited. Differences in immune system maturity, baseline laboratory reference ranges, hormonal influences, nutritional status, and environmental exposures may contribute to age-related variability in observed findings [6].

Such comparisons are of particular relevance in regions with a high burden of vitamin D deficiency and infectious diseases. Descriptive, age-stratified evaluations may help identify patterns that warrant further investigation and support the design of future longitudinal and interventional studies aimed at clarifying temporal and biological relationships [7,8].

Objective

The objective of this study was to describe and compare associations between vitamin D deficiency, selected immune markers, and reported infection patterns in pediatric and adult patients presenting to a tertiary care center.

## Materials and methods

Study design

This was a comparative, cross-sectional observational study conducted to describe and compare associations between vitamin D deficiency, selected immune markers, and reported infection patterns among pediatric and adult patients. The study design was chosen to allow age-stratified comparisons at a single time point without intervention or longitudinal follow-up.

Study setting and duration

The study was conducted at Avicenna Medical College and Hospital, Lahore. Data collection took place over a three-month period from 1 May 2025 to 31 July 2025. Clinical and laboratory data were obtained from both outpatient and inpatient departments.

Ethical approval

Ethical approval was obtained from the Avicenna Research Ethics Committee (IRB No. IRB-63/03/25/AVC, dated 21 April 2025). The study was conducted in accordance with the Declaration of Helsinki and national ethical guidelines. Written informed consent was obtained from all adult participants. For pediatric participants, assent was obtained where appropriate, along with written consent from parents or legal guardians. Participant confidentiality and data anonymity were strictly maintained.

Study population

The study population included pediatric patients aged 1-18 years and adult patients aged 19 years and above who presented to the hospital during the study period. Both male and female participants were included. Patients with chronic kidney disease, chronic liver disease, autoimmune disorders, malignancy, or those who had received vitamin D supplementation within the preceding three months were excluded to minimize major clinical confounding.

Participants were enrolled using a consecutive convenience sampling approach, whereby all eligible patients presenting during the data collection period were screened for inclusion.

Data collection

Demographic data, including age and sex, along with relevant clinical history, were collected using a structured data collection proforma. Venous blood samples were obtained under aseptic conditions for laboratory analysis.

Serum 25-hydroxyvitamin D (25(OH)D) levels were measured using a chemiluminescent immunoassay technique routinely employed in the hospital laboratory. Vitamin D deficiency was defined as serum 25(OH)D levels <20 ng/mL. Total leukocyte count and differential counts were analyzed using an automated hematology analyzer. Serum immunoglobulin levels (IgG, IgA, and IgM) were measured using nephelometric methods. C-reactive protein (CRP) was assessed using an immunoturbidimetric assay, and erythrocyte sedimentation rate (ESR) was measured using the Westergren method. All laboratory analyses were performed according to institutional standard operating procedures with internal quality control measures in place. Age-appropriate institutional reference ranges were used for the interpretation of immune markers.

Assessment of infection patterns

Reported infection patterns were assessed retrospectively based on documented medical records and structured patient or caregiver interviews covering the preceding six months. Respiratory and gastrointestinal infections were recorded if they required medical consultation or treatment during this period. This assessment was intended to describe reported infection frequency rather than to establish diagnostic severity or causality.

Statistical analysis

Data were entered and analyzed using the Statistical Package for the Social Sciences (SPSS) version 26.0 (IBM Corp., Armonk, NY, USA). Continuous variables were expressed as mean ± standard deviation, while categorical variables were presented as frequencies and percentages. Comparisons between pediatric and adult groups were performed using independent samples t-tests for continuous variables and chi-square tests for categorical variables. Pearson correlation analysis was used to assess associations between serum vitamin D levels and immune markers. All analyses were unadjusted and exploratory in nature. A p-value of less than 0.05 was considered statistically significant.

## Results

A total of 120 participants were included in the study, comprising 60 pediatric patients (aged 1-18 years) and 60 adult patients. Baseline demographic and clinical characteristics of both groups are summarized in Table 1. There was no statistically significant difference between the groups with respect to gender distribution (p=0.72). However, adult participants had a significantly higher mean BMI than pediatric participants (p<0.001).

**Table 1:** Baseline characteristics of participants Values are expressed as mean ± SD or frequency (%). An independent sample t-test was used for continuous variables and a chi-square (χ²) test for categorical variables. BMI: body mass index; SD: standard deviation.

Parameter	Pediatric (n=60)	Adult (n=60)	Test statistic	p-value
Age (years), mean ± SD	7.2±3.1	36.5±7.8	—	—
Male, n (%)	32 (53.3)	30 (50.0)	χ²=0.13	0.72
Female, n (%)	28 (46.7)	30 (50.0)	χ²=0.13	0.72
BMI (kg/m²), mean ± SD	16.8±2.4	24.3±3.5	t=13.4	<0.001

Disease susceptibility patterns differed between the two groups, as shown in Table 2. Recurrent respiratory infections were significantly more frequent among pediatric participants than adult participants (45% versus 20%, p=0.002). Gastrointestinal infections were also more common in children (p=0.01), whereas mild respiratory symptoms were more frequently observed in adults, although this difference did not reach statistical significance (p=0.23). These findings reflect differences in reported infection frequency between groups rather than confirmed diagnostic incidence or causal susceptibility.

**Table 2:** Disease susceptibility in vitamin D-deficient participants Values are expressed as frequency (%). Chi-square (χ²) test was used for group comparisons.

Disease Type	Pediatric (n=60)	Adult (n=60)	Test statistic	p-value
Recurrent respiratory infection	27 (45.0%)	12 (20.0%)	χ²=9.6	0.002
Gastrointestinal infection	15 (25.0%)	5 (8.3%)	χ²=6.6	0.01
Mild respiratory symptoms	10 (16.7%)	15 (25.0%)	χ²=1.4	0.23

Immune function parameters are presented in Table 3. Pediatric participants had lower mean total lymphocyte counts and IgG levels compared to adult participants, with these differences reaching statistical significance (p<0.001). No statistically significant differences were observed for IgA or IgM levels between the two groups. A significant positive correlation was observed between serum vitamin D levels and total lymphocyte count in both pediatric and adult participants (Pearson correlation coefficient r=0.52, p<0.001). This correlation represents an unadjusted association observed at a single time point.

**Table 3:** Immune function markers in pediatric versus adult participants Values are expressed as mean ± standard deviation. An independent sample t-test was applied.

Marker	Pediatric (n=60)	Adult (n=60)	Test statistic	p-value
Total lymphocyte count (×10⁹/L)	2.0±0.5	2.7±0.6	t=6.8	<0.001
IgG (g/L)	8.1±1.2	10.5±1.5	t=9.5	<0.001
IgA (g/L)	1.2±0.3	1.4±0.4	t=1.8	0.08
IgM (g/L)	1.0±0.2	1.1±0.3	t=1.6	0.12

Inflammatory markers were compared between the two groups and are detailed in Table 4. Mean CRP levels were significantly higher in pediatric participants than in adult participants (p<0.001). Similarly, ESR values were also higher among children (p=0.01).

**Table 4:** Inflammatory markers in pediatric versus adult participants Values are expressed as mean ± standard deviation. An independent sample t-test was used. CRP, C-reactive protein; ESR, erythrocyte sedimentation rate.

Marker	Pediatric (n=60)	Adult (n=60)	Test statistic	p-value
CRP (mg/L)	5.4±2.1	3.1±1.7	t=6.5	<0.001
ESR (mm/h)	15±6	12±5	t=2.6	0.01

## Discussion

This study evaluated associations between vitamin D deficiency, immune markers, and reported infection patterns in pediatric and adult participants. The findings showed that pediatric participants had lower measured serum vitamin D levels, differences in selected immune parameters, and higher reported frequencies of certain infections compared with adult participants. These findings represent descriptive associations observed at a single time point and should be interpreted within the context of the study’s cross-sectional design.

Lower total lymphocyte counts and IgG levels were observed in the pediatric group compared with adults. These findings are consistent with previous observational studies that have reported differences in immune markers among vitamin D-deficient children [9,10]. However, these between-group differences should be interpreted cautiously and in the context of normal developmental and biological variation between pediatric and adult immune systems, rather than as evidence of immunodeficiency or impaired immune function.

Several studies have reported associations between vitamin D deficiency and alterations in lymphocyte counts and immunoglobulin levels in children [11,12]. The present findings are in line with this literature, suggesting the coexistence of low vitamin D levels with differences in immune parameters during childhood. The observed differences between pediatric and adult participants likely reflect a combination of developmental immunology, nutritional status, and environmental exposures rather than differential biological sensitivity to vitamin D deficiency alone [13,14].

In adult participants, vitamin D deficiency was also associated with differences in immune markers, although these differences were less pronounced than those observed in children. Similar observations have been reported in adult populations, where vitamin D deficiency has been described alongside altered immune and inflammatory profiles without clear evidence of causality [15,16]. These findings underscore that vitamin D status may coexist with immune variation across age groups in observational settings.

Inflammatory markers, including CRP and ESR, were higher in pediatric participants compared with adult participants. Previous studies have reported associations between vitamin D deficiency and systemic inflammation, particularly in pediatric populations [17,18]. In the present study, these findings indicate differences in inflammatory marker levels between age groups but do not establish a causal relationship between vitamin D deficiency and inflammation.

A significant positive correlation was observed between serum vitamin D levels and total lymphocyte count in both pediatric and adult participants. This association is consistent with previous reports describing relationships between vitamin D status and immune cell counts [16]. However, this correlation was unadjusted and observed at a single time point; therefore, it does not establish a mechanistic or causal relationship and may be influenced by age, inflammatory status, or other unmeasured factors [19]. 

An important clinical observation in this study was the higher reported frequency of respiratory and gastrointestinal infections among pediatric participants. These findings reflect differences in reported infection patterns between age groups rather than confirmed diagnostic incidence or biological susceptibility. While vitamin D deficiency has been associated with infection frequency in several observational studies [20], the present findings should be regarded as hypothesis-generating and not indicative of a direct causal pathway.

Overall, this study contributes descriptive data on age-stratified associations between vitamin D deficiency, immune markers, and reported infection patterns in a tertiary care setting. The findings highlight the need for further longitudinal and interventional studies to clarify temporal relationships, assess potential effect modification by age, and determine whether correction of vitamin D deficiency influences immune or clinical outcomes.

Limitations

This study has several limitations. The cross-sectional design precludes assessment of temporality or causality between vitamin D status, immune markers, and reported infection patterns. The comparison between pediatric and adult groups was descriptive and exploratory, and immune parameters were evaluated using absolute laboratory values interpreted within age-appropriate reference ranges rather than age-standardized scores. Infection patterns were assessed retrospectively using medical records and participant or caregiver recall, which may be subject to recall and classification bias. Potential confounding factors such as dietary intake, sun exposure, socioeconomic status, vaccination history, and seasonal variation were not measured or adjusted for, and residual confounding cannot be excluded. As a single-center study with unadjusted analyses, the findings should be interpreted as associative and hypothesis-generating rather than definitive.

## Conclusions

In this cross-sectional observational study, vitamin D deficiency was found to be associated with differences in selected immune markers and reported infection patterns between pediatric and adult patients. Pediatric participants demonstrated lower measured serum vitamin D levels, differences in lymphocyte counts and IgG levels, and higher reported frequencies of certain infections compared with adults. These findings represent descriptive associations observed at a single time point and do not establish causality or directionality. The results highlight age-stratified patterns that warrant further investigation through longitudinal and interventional studies to clarify temporal relationships and determine whether modification of vitamin D status influences immune or clinical outcomes.

## References

[1] Wang L, Yu Y, Liu K, Yu X, Li Y (2025). The role of vitamin D in the prevention and treatment of acute respiratory infections in pediatric populations: a systematic review and meta-analysis of randomized controlled trials. BMC Pediatr.

[2] Ullah H (2025). Gut-vitamin D interplay: key to mitigating immunosenescence and promoting healthy ageing. Immun Ageing.

[3] Yang S, Yang W (2025). Correlation between serum vitamin D levels and immune function; clinical course in children with severe pneumonia: a single-center retrospective study. BMC Pediatr.

[4] Lazarus G, Putra IG, Junaidi MC, Oswari JS, Oswari H (2024). The relationship of vitamin D deficiency and childhood diarrhea: a systematic review and meta-analysis. BMC Pediatr.

[5] Wimalawansa SJ (2025). Vitamin D deficiency meets Hill's criteria for causation in SARS-CoV-2 susceptibility, complications, and mortality: a systematic review. Nutrients.

[6] Missilmani F, Maarabouni D, Salem-Sokhn E, Karras SN, Fakhoury HM, El Shamieh S (2025). Evaluation of vitamin D status, vitamin D receptor expression, and innate immune mediators in COVID-19. Front Endocrinol (Lausanne).

[7] D'Alessandro A, Ciavardelli D, Pastore A (2023). Contribution of vitamin D(3) and thiols status to the outcome of COVID-19 disease in Italian pediatric and adult patients. Sci Rep.

[8] Javadfar Z, Soltani S, Khamoushi F (2025). Effect of vitamin D supplementation on inflammatory status and behavioral symptoms in children with autism spectrum disorders: a double-blind randomized clinical trial. BMC Pediatr.

[9] Jeyakumar A, Bhalekar P, Shambharkar P (2024). Effect of vitamin D supplementation on the immune response to respiratory tract infections and inflammatory conditions: a systematic review and meta-analysis. Hum Nutr Metab.

[10] Garg D, Bhalla K, Nanda S, Gupta A, Mehra S (2024). Vitamin D status in children with community acquired pneumonia and its association with severity: a hospital-based study. Minerva Pediatr (Torino).

[11] Caliman-Sturdza OA, Gheorghita RE, Soldanescu I (2025). Vitamin D and COVID-19: clinical evidence and immunological insights. Life (Basel).

[12] Chan F, Cui C, Peng Y, Liu Z (2025). The associations among serum vitamin D concentration, systemic immune-inflammation index, and lifestyle factors in Chinese adults: a cross-sectional analysis. Front Nutr.

[13] Yarparvar A, Elmadfa I, Djazayery A, Abdollahi Z, Salehi F (2020). The association of vitamin D status with lipid profile and inflammation biomarkers in healthy adolescents. Nutrients.

[14] Alolayan A, Al-Wutayd O (2025). Association between vitamin D status and asthma control levels among children and adolescents: a retrospective cross-sectional study. BMC Pediatr.

[15] Trachtenberg T, Martirosyan D (2024). Addressing vitamin D deficiency through nutritional strategies: broader implications for immune health. Bioact Comp Health Dis.

[16] Bueno V (2023). Vitamin D, ageing, and the immune system. Explor Immunol.

[17] Carboo JA, Dolman-Macleod RC, Malan L, Lombard MJ (2024). High-dose oral vitamin D supplementation for prevention of infections in children aged 0 to 59 months: a systematic review and meta-analysis. Nutr Rev.

[18] Marusca LM, Reddy G, Blaj M (2023). The effects of vitamin D supplementation on respiratory infections in children under 6 years old: a systematic review. Diseases.

[19] Ismailova A, White JH (2022). Vitamin D, infections and immunity. Rev Endocr Metab Disord.

[20] Bikle DD (2022). Vitamin D regulation of immune function. Curr Osteoporos Rep.

